# Activation of monocytes and cytokine production in patients with peripheral atherosclerosis obliterans

**DOI:** 10.1186/1476-9255-8-23

**Published:** 2011-08-29

**Authors:** Camila R Corrêa, Luciane A Dias-Melicio, Sueli A Calvi, Sidney Lastória, Angela MVC Soares

**Affiliations:** 1 Departamento de Patologia, UNESP - Univ Estadual Paulista, Faculdade de Medicina - Campus Botucatu, CEP 18618-970, SP, Brasil; 2 Departamento de Doenças Tropicais e Diagnóstico por Imagem, UNESP - Univ Estadual Paulista, Faculdade de Medicina - Campus Botucatu, CEP 18618-970, SP, Brasil; 3 Departamento de Cirurgia e Ortopedia UNESP - Univ Estadual Paulista, Faculdade de Medicina - Campus Botucatu, CEP 18618-970, SP, Brasil; 4 Departamento de Microbiologia e Imunologia, UNESP - Univ Estadual Paulista, Instituto de Biociências - Campus Botucatu, CEP 18618-970, SP, Brasil

**Keywords:** Peripheral arteriosclerosis obliterans, monocytes, cytokines, peripheral blood

## Abstract

**Background:**

Arterial peripheral disease is a condition caused by the blocked blood flow resulting from arterial cholesterol deposits within the arms, legs and aorta. Studies have shown that macrophages in atherosclerotic plaque are highly activated, which makes these cells important antigen-presenting cells that develop a specific immune response, in which LDLox is the inducing antigen. As functional changes of cells which participate in the atherogenesis process may occur in the peripheral blood, the objectives of the present study were to evaluate plasma levels of anti-inflammatory and inflammatory cytokines including TNF-α, IFN-γ, interleukin-6 (IL-6), IL-10 and TGF-β in patients with peripheral arteriosclerosis obliterans, to assess the monocyte activation level in peripheral blood through the ability of these cells to release hydrogen peroxide (H_2_O_2_) and to develop fungicidal activity against *Candida albicans (C. albicans) in vitro*.

**Methods:**

TNF-α, IFN-γ, IL-6, IL-10 and TGF-β from plasma of patients were detected by ELISA. Monocyte cultures activated *in vitro *with TNF-alpha and IFN-gamma were evaluated by fungicidal activity against *C. albicans *by culture plating and Colony Forming Unit (CFU) recovery, and by H_2_O_2 _production.

**Results:**

Plasma levels of all cytokines were significantly higher in patients compared to those detected in control subjects. Control group monocytes did not release substantial levels of H_2_O_2 _*in vitro*, but these levels were significantly increased after activation with IFN-γ and TNF-α. Monocytes of patients, before and after activation, responded less than those of control subjects. Similar results were found when fungicidal activity was evaluated. The results seen in patients were always significantly smaller than among control subjects. *Conclusions: *The results revealed an unresponsiveness of patient monocytes *in vitro *probably due to the high activation process occurring *in vivo *as corroborated by high plasma cytokine levels.

## Background

Arterial peripheral disease is a condition caused by the blocking of blood flow as a result of cholesterol deposition in the arteries of the arms, legs and aorta [1].

Evidence suggests that low-density lipoprotein (LDL) modified by oxidation (LDLox) is the main triggering factor of the lesion. After the oxidation process, these particles become cytotoxic to endothelial cells, which once damaged, start to express and produce adhesion molecules and chemokines, leading to monocyte recruitment and adherence [2,3].

Studies have shown that macrophages in the atherosclerotic plaque are highly activated, followed by an increase in the expression of class II molecules of the major histocompatibility complex. This process makes macrophages important antigen-presenting cells for developing a specific immune response. In this case LDLox is the inducing antigen which causes a Th1 response, followed by production of interferon- gamma (IFN-γ) and tumor necrosis factor -alpha and -beta (TNF-α and TNF-β) and interleukin-12 (IL-12) [4-7]. Thus, IFN-γ has been considered one of the main cytokines released during atherosclerosis which, through an activation process of macrophages, amplifies the actions of these cells but in certain circumstances may lead to apoptosis [8].

Given the fact that the functional changes in the cells that participate in the atherogenesis process may occur in peripheral blood, the objectives of the present study were to evaluate the plasma levels of anti-inflammatory and inflammatory cytokines including interleukin-6 (IL-6), IFN-γ, IL-10 and transforming growth factor-beta (TGF-β) in patients with peripheral arteriosclerosis obliterans, to assess the level of monocyte activation in the peripheral blood through the ability of these cells to release hydrogen peroxide (H_2_O_2_) and to develop fungicidal activity against *Candida albicans (C. albicans) in vitro*.

## Patients and methods

### Patients

The present study was performed on ten male subjects, aged over 60 years, with moderate intermittent claudication, who were seen at the first time in the ambulatory of the Peripheral Vascular Surgery Service at the Clinic Hospital of the Botucatu Medical School - UNESP, Brazil. For this study both control subjects and patients were evaluated and excluded for presenting any of the following criteria: using medications, suffering from systemic arterial hypertension or any chronic disease, drinking alcohol or smoking.

The clinical diagnosis was performed by the ankle-brachial pressure index and exercise stress test. Using the same criteria, ten male subjects over 60 years old, with no peripheral arterial disease were also evaluated as the control group. All subjects were informed of the procedures and objectives of the study and signed a written informed consent. The study protocol was approved by the local Research Ethics Committee (653/00).

Blood samples collected both from patients and control subjects were placed in tubes containing heparin for biochemical analysis, cytokine measurement and the isolation and culturing of monocytes.

### Isolation of peripheral blood mononuclear cells

Heparinized venous blood samples were obtained from patients and healthy donors. Peripheral blood mononuclear cells (PBMC) were isolated by density gradient centrifugation at 400 g for 30 min on Ficoll-Paque™ Plus [density (d) = 1.077] (GE Healthcare Bio-Sciences AB, Uppsala). Briefly, 20 mL of heparinized blood was mixed with an equal volume of RPMI - 1640 tissue culture medium (Sigma-Aldrich, St. Louis, USA), and samples were layered over 10 mL of Ficoll-Paque™ Plus in a 50 mL conical plastic centrifuge tube. After centrifugation at 400 g for 30 min at room temperature, the interface layer of PBMC was harvested and washed twice with RPMI - 1640 tissue culture medium (Sigma-Aldrich). The PBMC suspension was stained with neutral red (0.02%) which is incorporated by monocytes and allows their identification and counting in a hemocytometer chamber. After counting, the suspension of mononuclear cells was adjusted to 2 × 10^6 ^monocytes/mL in RPMI-1640 (Sigma-Aldrich) containing 2 mM L-glutamine, 10% heat-inactivated human autologous serum, 20 mM HEPES and 40 μg/mL gentamicin (Complete Tissue Culture Medium - CTCM), dispensed at 100 μL/well in 96-well flat-bottomed plates (TPP, Trasadingen, Switzerland) and used for evaluation of fungicidal activity and H_2_O_2 _production. After incubation of cultures for 2 h at 37°C in 5% CO_2_, non-adherent cells were removed by aspiration and each well was rinsed twice with RPMI - 1640 tissue culture medium. The resulting monocyte cultures were treated with the following stimuli for 18 h at 37°C in 5% CO_2_: (i) CTCM, (ii) CTCM + IFN-γ human recombinant, or (iii) CTCM + TNF-α human recombinant (all from R&D Systems, Minneapolis, MN, USA) at different concentrations.

### Fungicidal activity of monocytes against *C. albicans*

Yeast cells of *C. albicans*, sample H-428/03, originally isolated from a patient of the Clinical Hospital of the Botucatu Medical School - UNESP, Brazil, and maintained by weekly subcultivation in yeast form at 35°C on BHI agar medium (Oxoid, Ltd.), were used after 5 or 6 days of growth.

After 18 h of incubation at 37°C in 5% CO_2_, supernatants from monocyte cultures, either activated with IFN-γ and TNF-α or not, were discarded and monocytes monolayers were challenged with 100 μL of *C. albicans *suspension containing 8 × 10^4 ^viable yeasts/mL (fungus-to-monocyte ratio of 1:25) prepared in CTCM plus 10% fresh autologous serum, as the source of complement for yeast opsonization, for 2.5 h at 37°C in 5% CO_2_. After the incubation period, culture supernatants were collected and the monocyte monolayers were washed several times with sterile distilled water to remove and to lyse monocytes with subsequent release of live fungi. Each well washing resulted in a final volume of 4.0 mL, and 0.1 mL was plated on brain-heart infusion (BHI) agar medium (OXOID). Inoculated plates, in triplicates of each culture, were incubated at 37°C, for 24 hours, in sealed plastic bags to prevent drying. After 10 days, the number of colony forming units (CFU) per plate was counted. The inoculum used for the challenge was also plated under the same conditions. The plates containing the material obtained from the monocyte-fungus cultures were labeled experimental plates and those with the inoculum alone were used as controls. Fungicidal activity percentage was determined by the following formula:

% Fungicidal Activity = [1 - (mean CFU recovered on experimental plates/mean CFU recovered on control plates)]×100

### Determination of hydrogen peroxide release (H_2_O_2_)

The production of H_2_O_2 _was determined according to the method described by Pick & Keisari (1980) and adapted by Pick & Misel (1981) [9,10]. After an 18-hour incubation period, supernatants of monocyte cultures, either activated or not with cytokines, were discarded and the cells were resuspended to the original volume in phenol red solution containing 140 mM of NaCl; 10 mM of phosphate buffered saline (pH 7) 5.5 mM of dextrose; 0.56 mM phenol red; 0.01 mg/mL of horseradish peroxidase, type II (Sigma, Chemical Co USA) and 1 ug of Phorbol Mirestate Acetate (PMA). Plates were incubated in a humidified chamber for 1 h in 5 % CO_2 _at 37°C. The reaction was then halted by the addition of 10 mL of 1 M NaOH and the absorbance at 620 nm was determined with a micro-ELISA reader (MD 5000, Dynatech Laboratories, Inc., Chantilly, VA., U.S.A.). Results were expressed as nanomoles of H_2_O_2_/2 × 10^5 ^cells using the standard curve established in each assay composed of known molar concentrations of H_2_O_2 _in phenol red buffer solution. In these experimental conditions, the curve was constructed based on these concentrations: 0.5; 1.0; 1.5 and 2.0 nM of H_2_O_2_.

### Plasma measurements of IL-6, TNF-α, IFN-γ, IL-10 and TGF-β

Plasma was separated from cell debris, by centrifuging at 1000 × *g *for 15 min, and stored at -70°C. The IL-6, TNF-α, IFN-γ, IL-10 and TGF-β concentrations were measured by capture ELISA using the Quantikine ELISA kit (R&D Systems, Minneapolis, MN, USA). ELISA was performed according to the manufacturer's protocol. Cytokine concentrations were determined according to a standard curve for serial two-fold dilutions of human recombinant cytokines. Absorbance values were measured at 492 nm using a micro-ELISA reader (MD 5000, Dynatech Laboratories). The lower limit of IL-6, TNF-α, IFN-γ, IL-10 and TGF-β detection was 5.0 pg/mL.

### Biochemical analyses

The analysis of cholesterol, triglycerides, HDL cholesterol, LDL cholesterol, glucose, urea, creatinine, alanine aminotransferase (ALT) and aspartate aminotransferase (AST) was performed using colorimetric enzymatic kits (CELM).

C-reactive protein (CRP) was measured by dry chemistry (Vitros 950 analyzer, Johnson & Johnson) and alpha-1-acid glycoprotein (α1-AGP) by nephelometry (Boehringer Nephelometer).

### Statistical Analysis

Monocyte activation-level results were evaluated by analysis of variance (ANOVA) for dependent samples, and mean values were compared using the Tukey-Kramer test for multiple comparisons [11].

Data from cytokines, biochemical evaluation, and lipid profile were assessed by the Student's *t *test.

All statistical analyses were performed using the software Graph Pad InStat 3.05 (Graph Pad Software, San Diego, CA, USA) at 5% significance level.

## Results

### Concentrations of plasma biochemical tests

Results on the plasma levels of glucose, alanine aminotransferase (ALT), aspartate aminotransferase (AST), urea and creatinine of patients and control subjects are shown in Table 1. Patients and control subjects presented levels within the normal range. Thus, no significant difference was found between patients and control subjects in the comparative analyses of all parameters evaluated. This finding shows that most subjects evaluated in this study did not show any sign suggestive of diabetes or of liver or kidney problems.

**Table 1:** Concentrations of plasma glucose, alanine aminotransferase, aspartate aminotransferase, urea, creatinine, total cholesterol, triglyceride, HDL cholesterol, LDL cholesterol, alpha 1-acid glycoprotein (α1- AGP) and C-reactive protein (CRP) presented by control subjects and patients.

	Control subjects	Patients
Glucose (mg/dL)	88.57 ± 2.46	92.42 ± 2.97
Alanine aminotransferase (U/L)	18.85 ± 2.76	20.42 ± 2.77
Aspartate aminotransferase (U/L)	16.57 ± 3.45	15.42 ± 3.77
Urea (mg/dL)	30.57 ± 2.53	36.14 ± 2.82
Creatinine (mg/dL)	1.1 ± 0.03	1.08 ± 0.05
Cholesterol (mg/dL)	201.4 ± 20.12	203.7 ± 20.32
Triglyceride (mg/dL)	107 ± 18	132 ± 22.4
HDL cholesterol (mg/dL)	54.0 ± 4.5	38.14 ± 3.2*
LDL cholesterol (mg/dL)	123.71 ± 6.9	163.14 ± 9.0*
α1-AGP (mg/dL)	76.71 ± 3.59	112.42 ± 8.71*
CRP (mg/dL)	0.10 ± 0	0.27 ± 0.60*

Normal values - glucose: 70-110 mg/dL; alanine transaminase: 30-65 U/L; aspartate transaminase: 15-37 U/L; urea: 15-40 mg/dL; creatinine: 0.6-1.4 mg/dL; cholesterol: < 200 mg/dL; triglyceride: < 200 mg/dL; HDL cholesterol: > 55 mg/dL; LDL cholesterol: < 100 mg/dL; α1-acid glycoprotein (α1-AGP): 30-120 mg/dL; C-reative protein (CRP): < 0.9 mg/dL. The results are expressed as mean ± SEM obtained from 10 patients and 10 control subjects. *p < 0.05 × Control Group

Results concerning total plasma cholesterol, triglyceride, HDL and LDL are also shown in Table 1. Therefore, total plasma cholesterol of both control subjects and patients was above normal levels for most subjects. Triglyceride levels of control subjects and patients were normal. HDL levels of most control subjects were normal, while all patients had levels outside of the normal range. LDL levels were observed to be significantly higher in patients than in control subjects.

Plasma levels of a1-AGP (a1-acid glycoprotein) and CRP (C-reactive protein) are also shown in Table 1. The analysis of these findings revealed higher levels of these two proteins in patients than in the control group.

### Plasma levels of pro and anti-inflammatory cytokines

Plasma levels of IL-6, TNF-α and IFN-γ were significantly higher in patients; the levels of the anti-inflammatory cytokines IL-10 and TGF-β revealed similar findings. Thus, plasma levels of the all cytokines analyzed were significantly higher in patients than in control subjects (Table 2).

**Table 2:** Concentrations of plasma cytokines (pro- and anti-inflammatory) in control subjects and patients.

	Control subjects	Patients
IL-6 (pg/mL)	9.23 ± 0.44	17.7 ± 1.7 *
TNF-α (pg/mL)	2.7 ± 0.3	5.0 ± 0.5*
IFN-γ (pg/mL)	181 ± 43.0	327 ± 70.0**
IL-10 (pg/mL)	10.0 ± 3.4	66.6 ± 14.3**
TGF-β (pg/mL)	105.6 ± 9.9	483.6 ± 161**

The results are expressed as mean ± SEM obtained from 10 patients and 10 control subjects. * p < 0.05 × Control Group; **p < 0.01 × Control Group

### Fungicidal activity of monocytes against *C. albicans*

**Figure 1:**
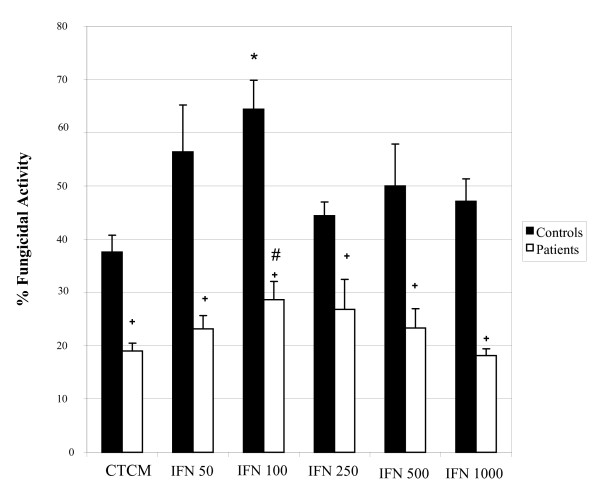
**Monocyte fungicidal activity against *Candida albicans***. Monocytes obtained from peripheral blood of control subjects and patients, before and after activation with different concentrations of interferon-gamma (IFN-γ), and after challenge with *C. albicans*. The results are expressed as mean ± SEM and derived from triplicate cultures of monocytes obtained from 10 patients and 10 control subjects. *p < 0.05 × Control (CTCM); #p < 0.05 × Patient (CTCM); + p < 0.05 × controls and patients in each group.

**Figure 2:**
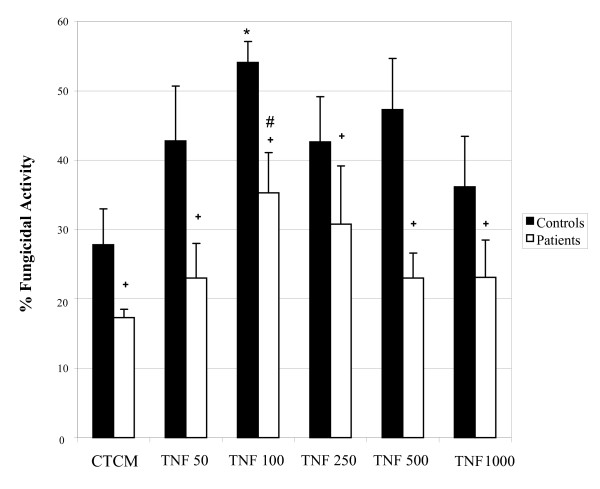
**Monocyte fungicidal activity against *Candida albicans***. Monocytes obtained from peripheral blood of control subjects and patients, before and after activation with different concentrations of tumoral necrosis factor-alpha (TNF-α), and after challenge with *C. albicans*. The results are expressed as mean ± SEM and derived from triplicate cultures of monocytes obtained from 10 patients and 10 control subjects. *p < 0.05 × Control (CTCM); #p < 0.05 × Patient (CTCM); + p < 0.05 × controls and patients in each group.

The ability of monocytes from control subjects and patients to develop fungicidal activity against *C. albicans in vitro *was evaluated before and after incubation with two cytokines involved in the activation process of these cells, namely IFN-γ and TNF-α. Results from IFN-γ and TNF-α are shown in Figures 1 and 2, respectively.

As to IFN-γ assays (Figure 1), non-activated monocytes of control subjects showed a significant fungicidal activity when compared to patients. The cytokine stimulation, especially at 100 units/mL, promoted a higher fungicidal activity when compared to non-activated cells. Monocytes of patients showed a response profile similar to that of control subjects, but with significantly lower response levels at all doses when compared to the ones from control subjects, before and after the activation process.

Similar findings were found in TNF-α assays (Figure 2); the non-activated monocytes of control subjects showed a significant fungicidal activity when compared to patients. The cytokine stimulation, mainly at 100 units/mL, promoted a higher fungicidal activity when compared to non-activated cells. Monocytes of patients also showed a response profile similar to that of control subjects when activated with TNF-α, but also with inferior response levels. Nevertheless, the results gathered from patients were always lower than those from the control group.

### Hydrogen peroxide (H_2_O_2_) production by activated monocytes

Similarly to the activation assays for evaluation of fungicidal activity, the levels of H_2_O_2 _released by monocytes from control subjects and patients were evaluated before and after IFN-γ and TNF-α activation.

**Figure 3:**
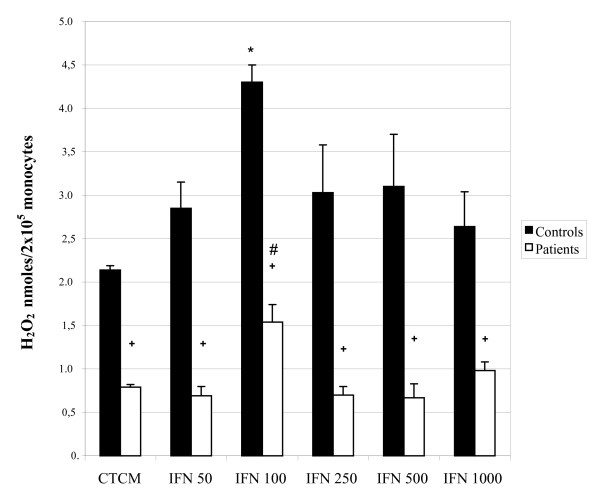
**Hydrogen Peroxide (H_2_O_2_) production by monocytes obtained from peripheral blood of control subjects and patients before and after activation with different concentrations of interferon - gamma (IFN-γ)**. The results are expressed as mean ± SEM and derived from triplicate cultures of monocytes obtained from 10 patients and 10 control subjects. *p < 0.05 × Control (CTCM); #p < 0.05 × Patient (CTCM); + p < 0.05 × controls and patients in each group.

**Figure 4:**
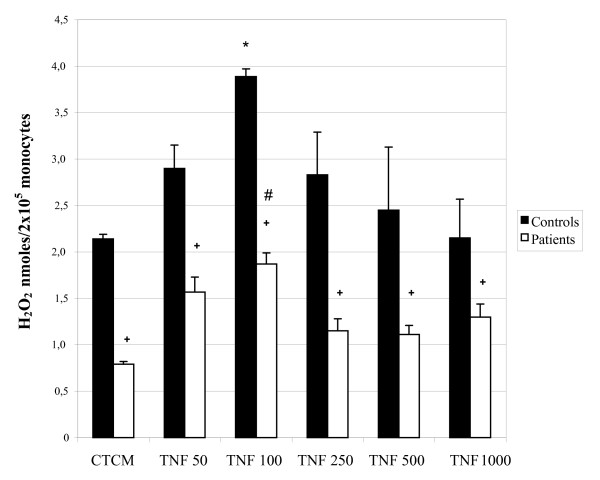
**Hydrogen Peroxide (H_2_O_2_) production by monocytes obtained from peripheral blood of control subjects and patients before and after activation with different concentrations of tumoral necrosis factor -alpha (TNF-α)**. The results are expressed as mean ± SEM and derived from triplicate cultures of monocytes obtained from 10 patients and 10 control subjects. *p < 0.05 × Control (MCCC); #p < 0.05 × Patient (CTCM); + p < 0.05 × control subjects and patients in each group.

In IFN-γ assays (Figure 3), non-activated monocytes of control subjects released substantial levels of the metabolite which increased significantly after activation, especially at the concentration of 100 U/mL, which agrees with the fungicidal activity assays. The response profile of monocytes from patients was similar to that of the control group, but with significantly lower levels found before and after the activation process. Similar findings were observed in TNF-α assays (Figure 4); the control and patient cells showed an increase in H_2_O_2 _levels after activation, mainly at the dose of 100 U/mL. However, levels of patients were always lower than those of the controls. Trials evaluating the ability to activate monocytes from control subjects and patients clearly showed that patients had significantly lower responses than those of controls, in all parameters tested.

## Discussion

In the present study patients with moderate intermittent claudication were evaluated. Results clarified the activation level of peripheral blood monocytes by analyzing fungicidal activity against *C. albicans *and hydrogen peroxide production *in vitro*, and through measuring TNF-α, IFN-γ, IL-6, IL-10 and TGF-β plasma levels. This is the first study to evaluate this process in patients with peripheral arteriosclerosis obliterans.

We also assessed the concentrations of plasma glucose, alanine aminotransferase, aspartate aminotransferase, urea, creatinine, total cholesterol, triglyceride, HDL cholesterol, LDL cholesterol, alpha 1-acid glycoprotein (α1- AGP) and C-reactive protein (CRP) to better characterize each patient's condition. Our results showed that patients had diminished HDL cholesterol and elevated LDL cholesterol, α1- AGP and CRP, in agreement with the literature [12-18].

Our results demonstrated that plasma levels of all pro-inflammatory cytokines analyzed - namely TNF-α, IFN-γ and IL-6 - were higher in these patients than those of the control group. These results are in accord with De Palma *et al*. [19] who demonstrated an increase in serum levels of these cytokines in patients with intermittent claudication. The authors suggested that a high level of cytokines is indicative of some complications such as claudication followed by myocardial infarction [19].

An increased serum level of some proinflammatory cytokines, such as TNF, has been detected in atherosclerotic events, including infarction and angina [20]. Nevertheless, Fiotti *et al*. [21], evaluating peripheral arterial disease, reported that receptors of these pro-inflammatory cytokines are more sensitive markers. The comparison between patients with intermittent claudication, with either ischemia or critical ischemia, and a control group revealed that TNF-α and IL-1 receptors were more significant markers than the cytokines themselves. However, more recent studies have shown an association between elevated TNF-α levels and peripheral arterial disease [19]. Thus, our results confirm the involvement of this cytokine in atherosclerotic processes. We observed that patients with higher levels of cytokines had more difficulty in the exercise stress test, reflecting the greater degree of ischemia, because these patients did not show any cardiac complication in contrast to reports from other authors [20,19].

Studies have also shown the role of IFN-γ in modulating the inflammatory response associated with atherosclerosis. Plasma levels of this cytokine are higher in patients with coronary diseases such as stable and unstable angina, and myocarditis [22]. The primary function of this cytokine is to activate monocytes/macrophages with a consequent increase of apoptosis, expression of adhesion molecules of the endothelium and synthesis of other pro-inflammatory cytokines such as IL-1 and IL-6.

Similar to results regarding pro-inflammatory cytokines, high levels of anti-inflammatory cytokines (IL-10 and TGF-β) were also found in patients evaluated in this study. These findings are in agreement with others that have shown the important anti-inflammatory function of IL-10 during atherosclerosis. This cytokine controls cell activation by decreasing the kappa B nuclear factor. Another function attributed to this cytokine in the atherosclerotic process is its ability to control excessive cell death by limiting the local inflammatory response. Findings from studies on atherosclerotic plaque of carotid arteries in rats confirm this function. Animals that received IL-10 presented decreases both in pro-inflammatory cytokine levels and in the apoptosis process, with the consequent stability of the atherosclerotic plaque [23,24].

Our evaluation of monocyte activation, through the analysis of fungicidal activity against *C. albicans *and hydrogen peroxide production *in vitro*, clearly showed that patients had significantly lower responses than those of the control group. These findings could be understood primarily as a diminished activation process in these patients. However, this idea was not confirmed by our findings showing high levels of both pro- and anti-inflammatory cytokines in patients' plasma. Several studies have reported that patients' cells are activated and consequently present higher capacity to release free radicals and to express MHC class II molecules, thus increasing their antigen presentation function. This process would lead to the development of a specific Th1 response [25]. Thus, our results showing low monocyte responses *in vitro *could be interpreted as a high state of activation of these cells *in vivo *in this stage of the disease, a process that would lead these cells to an exhausted condition, rendering them non-responsive *in vitro*. Similar studies found in the literature showed that foam cells isolated from aortas of hypercholesterolemic rats were capable of oxidizing lipoproteins, but not able to produce reactive oxygen species *in vitro *[26]. The authors suggested that this process may be related to intense phagocytosis and lipid uptake by monocytes, which would prevent them from responding to exogenous stimuli, with a consequent impairment of cell functions, such as a decrease in prostaglandin production *in vitro *[26,27].

In addition, natural control mechanisms of the inflammatory process in atherosclerosis, with a consequent inhibition of phagocyte activation have been described. This control may be related to anti-inflammatory actions of IL-10 and TGF-β. Genetically modified mice that are incapable of expressing LDL receptor, after receiving a hypercholesterolemic diet and transplantation of T cells, are able to produce high levels of IL-10, which is involved in diminishing the atherosclerotic process. The authors also reported that IL-10 acts through mechanisms towards a CD4^+ ^Th2 response that leads to an inhibition of the macrophage activation and the apoptosis process [8].

Briefly, this study allows us to suggest that monocytes from patients with aterosclerosis obliterans are highly activated *in vivo*. This process is probably responsible for triggering an intense inflammatory response, detected in these patients, that nevertheless appears to be controlled via the release of inflammatory cytokines such as IL-10. Further studies are being undertaken in our laboratory to elucidate these mechanisms.

## Competing interests

The authors declare that they have no competing interests.

## Authors' contributions

CRC performed all the experiments and drafted the manuscript. LADM participated in the experiments of dosage of hydrogen peroxide and fungicidal activity, helped with ELISA assays and was responsible for reviewing the manuscript. SAC participated in the performance of ELISA assays. SL was responsible for the medical screening. AMVCS conceived of the study, and participated in its design and coordination. All authors read and approved the final manuscript.
